# Protective Effects of Trimetazidine and Dexmedetomidine on Liver Injury in a Mesenteric Artery Ischemia–Reperfusion Rat Model via Endoplasmic Reticulum Stress

**DOI:** 10.3390/biomedicines12102299

**Published:** 2024-10-10

**Authors:** Sedat Ciftel, Tolga Mercantepe, Riza Aktepe, Esra Pinarbas, Zulkar Ozden, Adnan Yilmaz, Filiz Mercantepe

**Affiliations:** 1 Department of Gastroenterology and Hepatology, Erzurum Regional Education and Research Hospital, 25070 Erzurum, Turkey; sedat.ciftel@saglik.gov.tr; 2 Department of Histology and Embryology, Faculty of Medicine, Recep Tayyip Erdogan University, 53100 Rize, Turkey; tolga.mercantepe@erdogan.edu.tr (T.M.); zulkar.ozden@erdogan.edu.tr (Z.O.); 3 Department of Anatomy, Faculty of Medicine, Recep Tayyip Erdogan University, 53100 Rize, Turkey; riza.aktepe@erdogan.edu.tr; 4 Department of Biochemistry, Faculty of Medicine, Recep Tayyip Erdogan University, 53100 Rize, Turkey; esra.pinarbas@erdogan.edu.tr; 5 Department of Endocrinology and Metabolism Diseases, Faculty of Medicine, Recep Tayyip Erdogan University, 53100 Rize, Turkey

**Keywords:** dexmedetomidine, trimetazidine, endoplasmic reticulum stress, liver, mesenteric artery ischemia, ischemia–reperfusion injury

## Abstract

Background/Objectives: Acute mesenteric ischemia can lead to severe liver damage due to ischemia–reperfusion (I/R) injury. This study investigated the protective effects of trimetazidine (TMZ) and dexmedetomidine (DEX) against liver damage induced by mesenteric artery I/R via endoplasmic reticulum stress (ERS) mechanisms. Methods: Twenty-four rats were divided into four groups: control, I/R, I/R+TMZ, and I/R+DEX. TMZ (20 mg/kg) was administered orally for seven days, and DEX (100 µg/kg) was given intraper-itoneally 30 min before I/R induction. Liver tissues were analyzed for creatinine, alanine ami-notransferase (ALT), aspartate aminotransferase (AST), thiobarbituric acid reactive substances (TBARS), and total thiol (TT) levels. Results: Compared with the control group, the I/R group presented significantly increased AST, ALT, TBARS, and TT levels. TMZ notably reduced creatinine levels. I/R caused significant liver necrosis, inflammation, and congestion. TMZ and DEX treatments reduced this histopathological damage, with DEX resulting in a more significant reduction in infiltrative areas and vascular congestion. The increase in the expression of caspase-3, Bax, 8-OHdG, C/EBP homologous protein (CHOP), and glucose-regulated protein 78 (GRP78) decreased with the TMZ and DEX treatments. In addition, Bcl-2 positivity decreased both in the TMZ and DEX treatments. Conclusions: Both TMZ and DEX have protective effects against liver damage. These effects are likely mediated through the reduction in ERS and apoptosis, with DEX showing slightly superior protective effects compared with TMZ.

## 1. Introduction

Acute mesenteric ischemia is a frequently observed medical condition with a death rate ranging from 60% to 80% [1]. Possible causes of this condition include obstruction of the superior mesenteric artery (SMA), vascular surgeries in the abdomen and thorax, cardiopulmonary bypass, small bowel transplantation, or hemorrhagic shock [2]. This can result in significant local and remote tissue injury and subsequent malfunction of distant organs, particularly the liver and kidney [1]. Comprehending the mechanism that leads to distant organ damage resulting from acute mesenteric ischemia is crucial for reducing the occurrence of organ failure in survivors and enhancing outcomes following this injury.

A diverse range of clinical conditions can hinder blood flow to the intestines, including primary intestinal ailments and conditions affecting other organs or the entire body. Despite being limited in quantity, the literature has demonstrated that the polarization of Kupffer cells, oxidative stress, and inflammation are crucial factors in liver damage caused by small intestinal ischemia–reperfusion. This damage occurs because danger-related model substances are released from enterocytes into the portal circulation [1,2,3]. Nevertheless, the process remains incompletely understood. Consequently, there is currently no medicinal treatment available to prevent this illness.

Trimetazidine is a long-standing medication used to treat angina. Trimetazidine preserves cellular energy metabolism by preventing intracellular ATP reduction in cells exposed to hypoxia or ischemia [4]. Trimetazidine enhances glucose oxidation by decreasing the activity of long-chain 3-ketoacyl-CoA thiolase and suppressing the β-oxidation of fatty acids [5]. In an ischemic cell, the energy derived from glucose oxidation necessitates lower oxygen use than the β-oxidation pathway does. Increasing glucose oxidation improves cellular energy processes and helps maintain optimal energy metabolism during ischemia. Trimetazidine functions as a metabolic agent in individuals with ischemic heart disease, helping to sustain high-energy phosphate levels inside myocardial cells [6]. The elimination of the effects of ischemia is accomplished without concurrent hemodynamic consequences [7].

Dexmedetomidine is a medication that activates alpha-2 adrenergic receptors and has sedative, analgesic, and anxiolytic effects. It was initially created for use in intensive care units [8]. Nevertheless, the protective effects of dexmedetomidine against ischemia–reperfusion injury have prompted additional research into the potential therapeutic applications of this drug, as demonstrated by various experimental and clinical studies [9,10,11,12,13]. Dexmedetomidine binds to alpha-2 adrenergic receptors in the central nervous system [14]. Dexmedetomidine has a notable benefit in that it does not induce respiratory depression, which sets it apart from benzodiazepines [15]. Dexmedetomidine has demonstrated efficacy in mitigating ischemia–reperfusion injury in vital organs, including the heart, kidney, and liver [9,15,16,17]. While there is evidence that it can decrease oxidative stress and inflammation by enhancing antioxidant defense mechanisms, the specific underlying mechanism remains unclear.

The endoplasmic reticulum (ER) is a ubiquitous organelle in eukaryotic cells that plays a crucial role in protein synthesis, folding, and secretion [18]. Any adverse alteration in the environment that disturbs the balance within cells, such as oxidative stress, can impact the normal functioning of the endoplasmic reticulum (ER), leading to a condition known as ER stress (ERS) [19]. ERS triggers reaction mechanisms to increase the ability to fold proteins or eliminate misfolded proteins. These response mechanisms are managed through a set of primary pathways known as the “unfolded protein response (UPR)” [20]. The proteins C/EBP homologous protein (CHOP) and glucose-regulated protein 78 (GRP78) are essential in this process [21]. The expression level of GRP78 has been acknowledged as an indicator of ERS, and the ability of the ER to recover CHOP may govern cell apoptotic signaling pathways that are activated by excessive and prolonged ERS [22]. During ERS, the protein GRP78 attempts to alleviate the load caused by misfolded proteins, whereas the protein CHOP initiates programmed cell death (apoptosis) during excessive ERS [23]. These two proteins have significant functions in deciding the destiny of cells. GRP78 functions as a cell protector and regulates the activation of ERS sensors by binding to them. CHOP is involved in initiating apoptosis. ERS is a significant factor in the development of many disorders [23,24,25].

This study aimed to examine the protective effects of trimetazidine and dexmedetomidine against liver injury induced by mesenteric artery ischemia–reperfusion. Research has assessed whether these effects are facilitated by processes of endoplasmic reticulum stress, oxidative stress, and apoptotic pathways.

## 2. Materials and Methods

The animal studies were conducted in compliance with the guidelines outlined in the “Guide for the Care and Use of Laboratory Animals” [26]. This study was also approved by the Recep Tayyip Erdoğan University Local Ethics Committee for Experimental Animals, with an approval date of 23 February 2024 and an approval number of 2024/06.

### 2.1. Experimental Animals and Groups

This investigation utilized a cohort of 24 male Sprague–Dawley rats aged 3–4 months and weighing approximately 250–300 g. The animals were kept in a chamber at a constant temperature of 22–25 °C. The room had a 12 h cycle of light and darkness. The animals were given seven days to become acquainted with the room before they were used for the experiments. The rats were fed a regular diet consisting of pellets and had unrestricted access to water. Prior to surgery, the animals were fasted for 12 h while having unrestricted access to water. The number of animals in our study groups was determined according to the methods of Arifin, Charan, and Allgoewer et al. [27,28,29]. The rats were allocated into four groups via random assignment, ensuring that there were six animals in each group:

Group 1 (control group): Rats that did not receive any intervention.

Group 2 (ischemia–reperfusion (I/R) group): This group was subjected to ischemia, a lack of blood supply, for 1 h. This was followed by a period of reperfusion, which is the restoration of blood flow, and lasted 1 h. The procedure was carried out by clamping the superior mesenteric artery, as described by Gonzalez et al. in 2015 [30].

Group 3 (I/R+trimetazidine (I/R+TMZ) group): Prior to inducing small bowel ischemia–reperfusion injury by clamping the superior mesenteric artery, a daily oral gavage of 20 mg/kg trimetazidine was given for seven days [6,31]. The most recent administration of trimetazidine occurred 1 h before the induction of mesenteric artery ischemia.

Group 4 (the I/R+dexmedetomidine (I/R+DEX) group): A single dose of 100 µg/kg dexmedetomidine was given intraperitoneally 30 min before the induction of ischemia–reperfusion injury [32,33,34].

### 2.2. Experimental Procedures

Before the experimental procedures commenced, all the animals were fasted for 12 h. The rats in groups 2, 3, and 4, subjected to ischemia–reperfusion injury, were placed in the supine position on the operating table and placed under a heat lamp. They were administered general anesthesia consisting of 50 mg/kg ketamine and 10 mg/kg xylazine [35]. Gonzalez et al. (2015) reported that the superior mesenteric artery was clamped by an incision in the abdominal midline [30]. For 1 h, the artery was constricted to induce ischemia. The clamps were subsequently withdrawn, and reperfusion was allowed for another hour, as described by Şahin et al. in 2013 [33]. The degree of reperfusion was determined by visually observing the intestines transitioning from purple to the usual pink color.

### 2.3. Chemicals and Drugs

Trimetazidine (Vastarel 20 mg, Servier İlaç ve Araştırma A.Ş., Maslak/Istanbul, Türkiye), DEX (Precedex 200 mcg, 2 mL flask, Hospira Inc., North Rocky Mount, MC, USA), ketamine HCL (Ketalar 500 mg, Pfizer İlaçları Ltd. Sti., Istanbul, Türkiye), and xylazine (Rompun 2%, Bayer, Istanbul, Türkiye) were employed in the study.

### 2.4. Biochemical Analysis

A solution of 20 mM sodium phosphate and 140 mM potassium chloride was prepared, with a pH of 7.4, in a volume of 1 L. Subsequently, 1 mL of homogenization solution was introduced to 100 mg of tissue, and the liver tissue was homogenized via a homogenizer (QIAGEN Tissue Lyser II, Hilden, Germany), followed by centrifugation at 800× *g* for 10 min at 4 °C. Assays for total thiols (TTs) and thiobarbituric acid reactive substances (TBARS) were conducted using the acquired supernatant [36].

To measure the levels of malondialdehyde (TBARS, MDA) in the tissue, a 200 μL liver tissue sample was combined with 50 μL of sodium dodecyl sulfate (8.1%, *w*/*v*), 375 μL of thiobarbituric acid (0.8%, *w*/*v*), and 375 μL of acetic acid (20%, *v*/*v*). The mixture was then placed in a boiling water bath for 1 h. Next, the samples were centrifuged at 750× *g* for 10 min. A spectrophotometric measurement was subsequently taken at a wavelength of 532 nm. The results were quantified in millimoles per liter (mmol/L) [37].

To measure the amount of glutathione in liver tissue, a 42 μL sample was combined with 42 μL of Ellman reagent and 117 μL of disodium hydrogen phosphate (0.3 M). The formed yellow complex was measured via spectrophotometry at a wavelength of 412 nm. The results were quantified in millimoles per liter [38].

The AU680 Beckman Coulter apparatus (Beckman Coulter, Inc., Brea, CA, USA) was used to perform tests for creatinine (mg/dL), alanine aminotransferase (ALT, (U/L)), and aspartate aminotransferase (AST (U/L)). The tests were conducted via Beckman brand kits following the manufacturer’s instructions.

### 2.5. Histopathological Analysis

The liver tissue samples were preserved in 10% neutral formalin and subjected to standard histological methods for further examination. Sections measuring 4–5 micrometers in thickness were extracted from liver tissue samples fixed in paraffin. These sections were then stained with hematoxylin and eosin (H&E) to facilitate analysis via a light microscope. The liver tissue was evaluated for histopathological damage via the Liver Histopathological Damage Score (LHDS). The scoring was based on the presence of intralobular and perilobular necrosis, vascular congestion, and periportal inflammation, following the methods used in ischemia and reperfusion studies [39,40]. Two histopathologists blinded to the study groups thoroughly examined 25 randomly chosen regions from each rat’s five randomly selected liver slides via light microscopy. Histopathological damage scoring of the liver was performed by evaluating the presence of hepatocellular hydropic degeneration, intralobular necrosis, interlobular necrosis, perilobular inflammation, and vascular congestion, which were graded as absent = 0 (≤5%), mild = 1 (≤25%), moderate = 2 (≤50%), and severe = 3 (≤75%), (Supplementary Table S1) [32].

### 2.6. Immunohistochemical (IHC) Analysis

To perform the IHC technique, thin sections measuring 1–2 μm were extracted from paraffin blocks of liver tissue and mounted on slides with a positive charge. The removal of paraffin was then carried out. The liver tissue slices were analyzed via immunohistochemistry labeling with primary antibodies against CHOP (E-AB-70087, 1/300, Elabscience, Houston, TX, USA), GPR78 (SC-13539, 1/350, Santa Cruz Biotechnology Inc. Dallas, TX, USA), 8-hydroxy-2′-deoxyguanosine (8-OHdG) (Santa Cruz, SC-66036, 1/200, Santa Cruz Biotechnology Inc., Dallas, TX, USA), cleaved caspase-3 (Abcam, ab4051, 1/100), anti-Bax ab32503, 1/200, (Abcam, Cambridge, UK), and anti-Bcl-2 (Abcam, ab32124, 1/200). After exposure to primary antibodies, the liver tissues were subsequently incubated with a secondary antibody (goat anti-rabbit IgG H&L HRP, ab205719; Abcam, Cambridge, UK). The image signal was acquired via a light microscope by applying a diaminobenzidine chromogen (Ultraview, Leica Biosystems, Nussloch, Germany) to the tissues. The sample was counterstained with Harris hematoxylin and then protected with a suitable sealing solution.

Two histopathologists who were unaware of the study groups assessed the number of positively stained cells in liver tissue sections exposed to primary antibodies against Caspase-3, Bax, Bcl-2,8-OHdG, CHOP, and GRP-78. Semiquantitative analysis of liver IHC staining was performed by evaluating immunopositivity in hepatocytes as absent = 0 (≤5%), mild = 1 (≤25%), moderate = 2 (≤50%), and severe = 3 (≤75%), (Supplementary Table S2). The positive IHC score was determined by randomly selecting 25 distinct regions from each of the five separate preparations of rats [29].

### 2.7. Statistical Analysis

The study data were analyzed via SPSS version 20 software (IBM Corp., Armonk, NY, USA). The Shapiro–Wilk test, Q–Q plot, skewness–kurtosis test, and Levene’s test were utilized to assess the normality of the data. These tests were performed on nonparametric data, and the results are reported as median values with interquartile ranges (25–75%). Intergroup comparisons were conducted via the Kruskal–Wallis test, followed by pairwise Mann–Whitney U test comparisons with a Dunn–Bonferroni adjustment. *p* values less than 0.05 were considered statistically significant.

## 3. Results

### 3.1. Results of the Biochemical Analysis

The results of the comparative biochemical analysis of the creatine, AST, ALT, TBARS, and TT levels among the experimental groups are presented in Table 1. In summary, the AST, ALT, TBARS, and TT levels were substantially greater in the I/R, TMZ, and DEX groups than in the control group. However, the increase was particularly apparent in the I/R group (*p* < 0.001, *p* = 0.033, *p* = 0.014, *p* = 0.039, *p* = 0.018, *p* = 0.034, *p* = 0.038, *p* = 0.008, respectively). Nevertheless, the level of creatine in the TMZ group was similar to that in the control group and considerably lower than that in the I/R group (*p* = 0.006). Although the DEX group presented a substantial increase in creatine levels compared with those of the control group (*p* = 0.027), there was no statistically significant difference between the DEX group and the I/R group.

### 3.2. Histopathological Analysis

**Figure 1:**
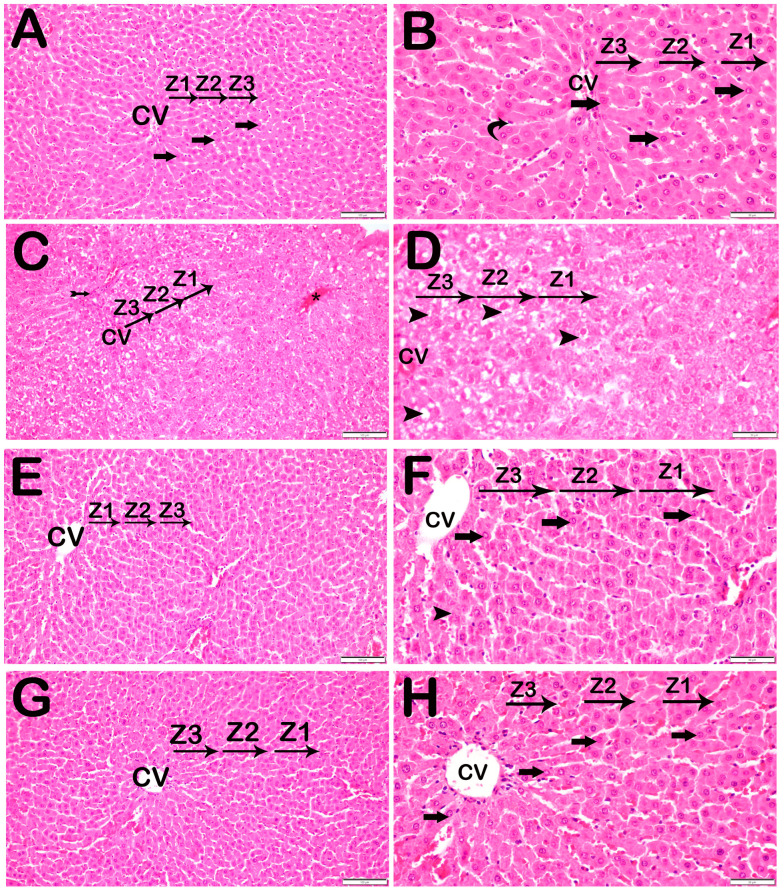
Representative light microscopic screen image of H&E-stained liver tissue sections. Central vein (CV). (**A**) (×20) and (**B**) (×40). Control group: In the liver tissue sections belonging to the control group, Remark cords containing hepatocytes with a normal structure (arrow) were observed (LHDS: 0(0-1)). (**C**) (×20) and (**D**) (×40). I/R group: In the liver tissue, widespread necrotic hepatocytes (arrowhead) are observed, primarily in Zone 1 (Z1) of the intralobular areas. Additionally, necrotic hepatocytes (arrowhead), vascular congestion (asteriks), and infiltrative areas (tailed arrow) are observed in the perilobular regions. (LHDS: 9(8-10)). (**E**) (×20) and (**F**) (×40). I/R+TMZ group: In the liver tissue, a reduction in intralobular necrosis, particularly perilobular necrosis, was observed. Additionally, a decrease in periportal infiltrative areas and vascular congestion was noted (LHDS: 4(3-5)). (**G**) (×20) and (**H**) (×40). I/R+DEX group: In the liver tissue, a decrease in necrotic hepatocytes was observed in both the intralobular and perilobular areas. Additionally, a reduction in periportal infiltrative areas and vascular congestion was noted (LHDS: 2(1-3)).

The histological investigation involved examining findings such as liver cell necrosis, infiltration of inflammatory cells, vascular congestion regions, and hepatic lobule integrity. The liver tissue slices of the control group did not exhibit any abnormal signs. One hour after blood flow was restored, the rats in the I/R group exhibited increased death of liver cells, particularly in Zone 1, as well as infiltration of cells around the lobules and congestion of blood vessels in wider areas. However, the rats in the I/R+TMZ group and I/R+DEX group exhibited normal conditions, similar to those in the control group. The rats in the I/R+TMZ and I/R+DEX groups presented necrotic hepatocytes in the intralobular and perilobular areas, similar to those in the control group. However, we found that the I/R+DEX group had significantly fewer infiltrative areas and vascular congestion in the perinobular areas than did the I/R+TMZ group (Figure 1, Table 2).

### 3.3. Immunohistochemical (IHC) Analysis

An examination of Caspase-3-, Bax-, Bcl-2-, 8-OHdG-, CHOP-, and GRP 78-positive scores in liver tissue sections revealed notable differences across the different groups.

**Figure 2:**
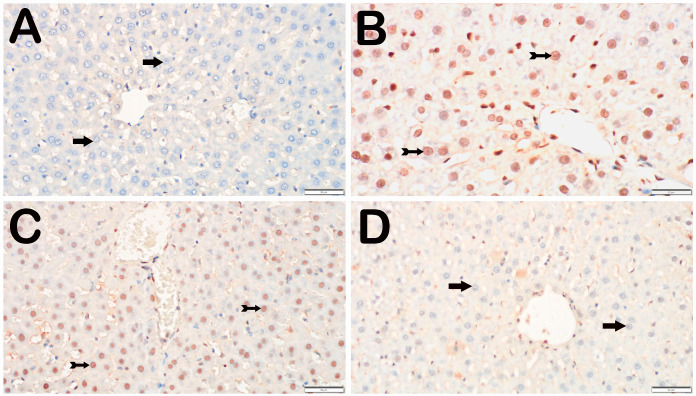
Representative light microscopic screen images of liver tissue sections incubated with a Caspase-3 primary antibody. (**A**) (×20). Control group: In the liver tissue sections from the control group, hepatocytes with a normal structure (arrow) were immunonegative (caspase-3 positivity score: 0(0-0)). (**B)** (×20). I/R group: In the liver tissue, hepatocytes showing intense Caspase-3 immunopositivity (spiral arrow) are observed, primarily in Zone 1 of the intralobular areas (Caspase-3 positivity score: 1(1-2)). (**C**) (×20). I/R+TMZ group: In the liver tissue, a decrease in the number of hepatocytes positive for Caspase-3 was observed, particularly in the intralobular and perilobular areas (Caspase-3 positivity score: 0.5(0-1)). (**D**) (×20). I/R+DEX group: A decrease in the number of apoptotic hepatocytes positive for Caspase-3 in the intralobular and perilobular areas was observed, with a widespread presence of immunonegative hepatocytes (caspase-3 positivity score: 0(0-1)).

With respect to the presence of Caspase-3, the control group did not have any hepatocytes that were positive for Caspase-3. Compared with the control group, the I/R group presented a substantial increase in the number of hepatocytes positive for Caspase-3 (*p* = 0.001). Compared with the I/R group, the I/R+TMZ group presented a notable reduction in the number of hepatocytes positive for Caspase-3 (*p* = 0.002). Furthermore, there was a notable decrease in the number of hepatocytes positive for Caspase-3 in the I/R+DEX group compared with the I/R group (*p* = 0.002) (Table 3, Figure 2).

**Figure 3:**
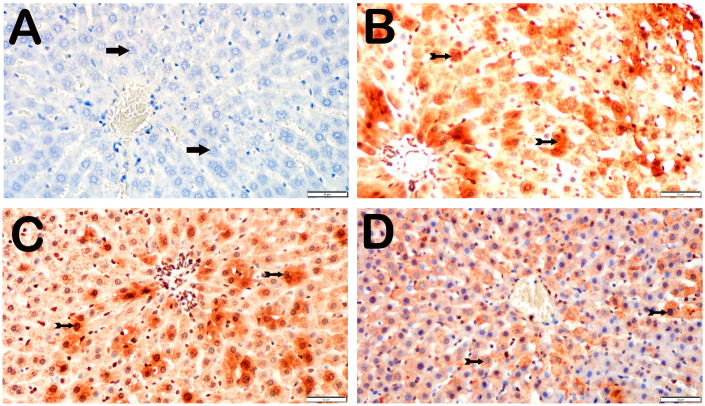
Representative light microscopic screen images of liver tissue sections incubated with 8-OHdG primary antibody. (**A**) (×20). Control group: In the liver tissue sections from the control group, hepatocytes with a normal structure (arrow) were 8-OHdG-negative (8-OHdG positivity score: 0(0-0)). (**B**) (×20). I/R group: In the liver tissue, hepatocytes showing intense immunopositivity with 8-OHdG primary antibody (spiral arrow) are observed, primarily in Zone 1 of the intralobular areas (8-OHdG positivity score: 2(2-3)). (**C**) (×20). I/R+TMZ group: A decrease in hepatocytes showing 8-OHdG immunopositivity was observed in the intralobular and perilobular areas (8-OHdG positivity score: 1(1-2)). (**D**) (×20). I/R+DEX group: A decrease in hepatocytes showing intense 8-OHdG immunopositivity in the intralobular and perilobular areas was observed, with a widespread presence of immunonegative hepatocytes (arrow, 8-OHdG positivity score: 1(0-1)).

With respect to the presence of 8-OHdG, no hepatocytes in the control group were positive for 8-OHdG. Compared with the control group, the I/R group presented a substantial increase in the number of apoptotic hepatocytes that were positive for intense 8-OHdG, particularly in Zones 1, 2, and 3 (*p* = 0.001). Compared with the I/R+TMZ group, the I/R+TMZ group presented a notable reduction in 8-OHdG-positive hepatocytes (*p* = 0.002). Furthermore, the number of hepatocytes positive for 8-OHdG was considerably lower in the I/R+DEX group than in the I/R group (*p* = 0.002). The quantity of 8-OHdG-positive hepatocytes in the I/R+DEX group was significantly lower than that in the I/R+TMZ group (*p* = 0.001) (Table 3, Figure 3).

**Figure 4:**
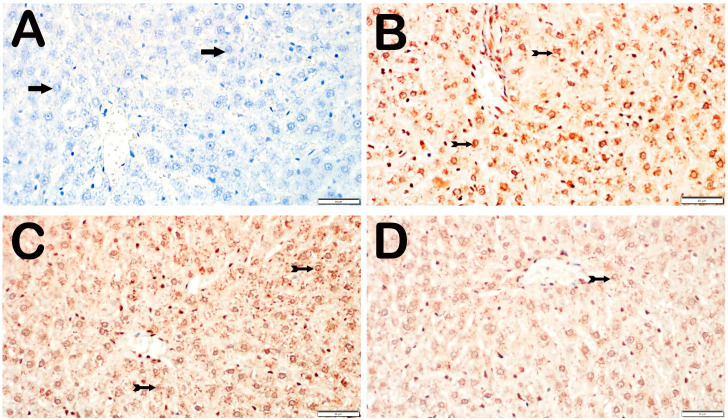
Representative light microscopic screen images of liver tissue sections incubated with CHOP primary antibody. (**A**) (×20). Control group: In the liver tissue sections from the control group, hepatocytes with a normal structure (arrow) were observed to be CHOP-negative (CHOP positivity score: 0(0-0)). (**B**) (×20). I/R group: In the intralobular areas, primarily in Zone 1, hepatocytes showing intense CHOP positivity (spiral arrow) are observed (CHOP positivity score: 2(1-2)). (**C**) (×20). I/R+TMZ group: A decrease in hepatocytes showing CHOP positivity was observed in the perinobular areas, particularly in the intralobular regions (CHOP positivity score: 1(0-1)). (**D**) (×20). I/R+DEX group: A decrease in hepatocytes showing intense immunopositivity in the intralobular and perilobular areas was observed, with a widespread presence of CHOP-negative hepatocytes (arrow, CHOP positivity score: 0.5 (0-1)).

With respect to CHOP positivity, the control group did not exhibit any hepatocytes that tested positive for CHOP. Compared with those in the control group, the number of CHOP-positive hepatocytes in the I/R group was substantially greater (*p* = 0.001). Compared with those in the I/R group, the number of CHOP-positive hepatocytes in the I/R+TMZ group was notably lower (*p* = 0.023). A notable decrease in the number of CHOP-positive hepatocytes was observed in the I/R+DEX group compared with the I/R group, and fewer CHOP-positive hepatocytes were observed in the I/R+DEX group than in the I/R+TMZ group (*p* = 0.001) (Table 3, Figure 4).

**Figure 5:**
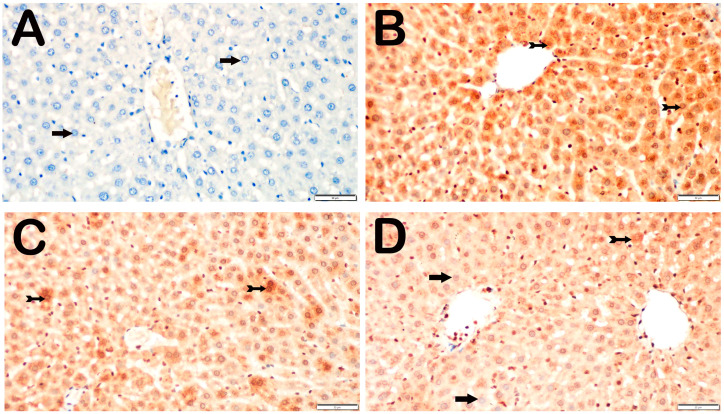
Representative light microscopy images of liver tissue sections incubated with an anti-GRP78 primary antibody. (**A**) (×20). Control group: In the liver tissue sections from the control group, hepatocytes with a normal structure (arrow) were observed to be GRP78-negative (GRP78 positivity score: 0(0-0)). (**B**) (×20). I/R group: In the intralobular areas, primarily in Zone 1, hepatocytes showing intense GRP78 positivity (spiral arrow) are observed (GRP78 positivity score: 2(2-2)). (**C**) (×20). I/R+TMZ group: A decrease in hepatocytes showing GRP78 positivity was observed in the perilobular areas, particularly in the intralobular regions (GRP78 positivity score: 1.5(1-2)). (**D**) (×20). I/R+DEX group: A decrease in hepatocytes showing intense immunopositivity in the intralobular and perilobular areas was observed, with a widespread presence of GRP78-negative hepatocytes (arrow, GRP78 positivity score: 1(0-1)).

With respect to the presence of GRP 78, no hepatocytes in the control group tested positive for GRP 78. Compared with the control group, the I/R group presented a substantial increase in the number of GRP 78-positive hepatocytes (*p* = 0.001). Compared with the I/R group, the I/R+TMZ group presented a notable reduction in the number of hepatocytes positive for GRP 78 (*p* = 0.007). The number of GRP 78-positive hepatocytes was considerably lower in the I/R+DEX group than in the I/R group (*p* = 0.002) (Table 3, Figure 5).

**Figure 6:**
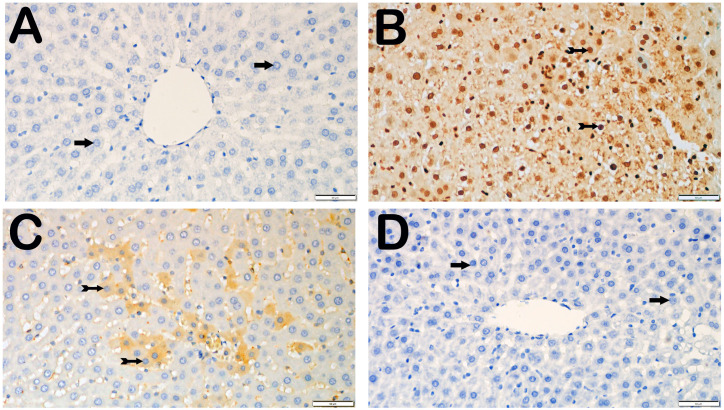
Representative light microscopy images of liver tissue sections incubated with a Bax primary antibody. (**A**) (×20). Control group: In the liver tissue sections from the control group, hepatocytes with a normal structure (arrow) were observed to be Bax-negative (Bax positivity score: 0(0-0)). (**B**) (×20). I/R group: In the intralobular areas, which are primarily in Zone 1, hepatocytes showing intense Bax positivity (spiral arrow) are observed (Bax positivity score: 2(2-3)). (**C**) (×20). I/R+TMZ group: A decrease in hepatocytes showing Bax positivity was observed in the perilobular areas, particularly in the intralobular regions (Bax positivity score: 1(1-1.5)). (**D**) (×20). I/R+DEX group: A decrease in hepatocytes showing intense immunopositivity in the intralobular and perilobular areas was observed, with a widespread presence of Bax-negative hepatocytes (arrow, Bax positivity score: 0(0-1)).

With respect to the presence of Bax, no hepatocytes in the control group tested positive for Bax. Compared with the control group, the I/R group presented a substantial increase in the number of Bax-positive hepatocytes (*p* = 0.001). Compared with the I/R group, the I/R+TMZ group presented a notable reduction in the number of hepatocytes positive for Bax (*p* = 0.001). The number of Bax-positive hepatocytes was considerably lower in the I/R+DEX group than in the I/R group (*p* = 0.001). In addition, compared with the I/R+TMZ group, the I/R+DEX group presented a notable reduction in the number of hepatocytes positive for Bax (*p* = 0.01) (Table 3, Figure 6).

**Figure 7:**
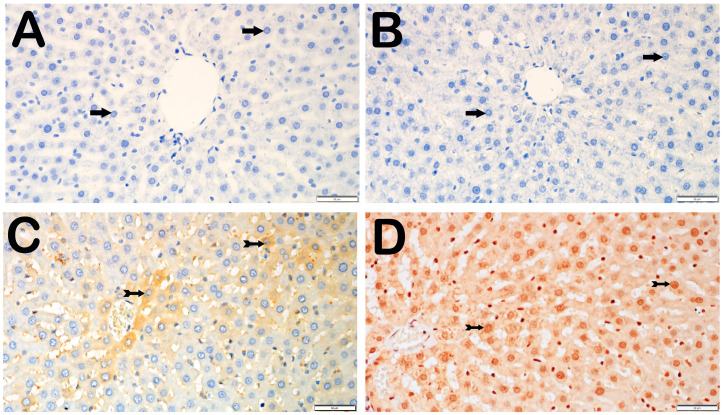
Representative light microscopy images of liver tissue sections incubated with a Bcl-2 primary antibody. (**A**) (×20). Control group: In the liver tissue sections from the control group, hepatocytes with a normal structure (arrow) were observed to be Bcl-2-negative (Bcl-2 positivity score: 0(0-0)). (**B**) (×20). I/R group: In the intralobular areas, primarily in Zone 1, hepatocytes showing Bcl-2 negativity (spiral arrow) are observed (Bcl-2 positivity score: 0(0-1)). (**C**) (×20). I/R+TMZ group: An increase in hepatocytes showing Bcl-2 positivity was observed in the perilobular areas, particularly in the intralobular regions (Bcl-2 positivity score: 1(1-2)). (**D**) (×20). I/R+DEX group: An increase in hepatocytes showing intense immunopositivity in the intralobular and perilobular areas was observed, with a widespread presence of Bcl-2-positive hepatocytes (arrow, Bcl-2 positivity score: 2(2-3)).

With respect to the presence of Bcl-2, no hepatocytes in the control group tested positive for Bcl-2. Similarly, the I/R group presented with Bcl-2 immunonegative hepatocytes. On the other hand, compared with the I/R group, the I/R+TMZ group presented a notable increase in the number of hepatocytes positive for Bcl-2 (*p* = 0.001). Similarly, the number of Bcl-2-positive hepatocytes was considerably greater in the I/R+DEX group than in the I/R group (*p* = 0.001). In addition, compared with the I/R+TMZ group, the I/R+DEX group presented a notable increase in the number of hepatocytes positive for Bcl-2 (*p* = 0.001) (Table 3, Figure 7).

These findings indicate that both the TMZ and DEX treatments effectively decreased indicators of cell death, endoplasmic reticulum stress, and oxidative damage caused by ischemia/reperfusion injury in liver tissue. However, DEX had a somewhat more pronounced effect than TMZ on reducing the number of hepatocytes positive for 8-OHdG and CHOP.

## 4. Discussion

This study investigated the impact of trimetazidine and dexmedetomidine on the liver in a superior mesenteric artery ischemia–reperfusion model. The results of our investigation demonstrated that both medicines effectively decrease the degree of apoptosis, oxidative stress, and endoplasmic reticulum stress that occur as a result of ischemia/reperfusion injury in liver tissue. Nevertheless, the outcomes derived from biochemical analyses do not entirely align with these observations.

Our biochemical analyses revealed substantial increases in creatine, AST, ALT, and TBARS levels and a decrease in TT levels in the I/R group compared with those in the control group, as anticipated. Furthermore, the compromised structural integrity of the liver, the existence of necrotic cells, extensive inflammation, and congested areas found in the I/R group provide further evidence that distant organs, such as the liver and kidney, are similarly impacted in cases of mesenteric artery ischemia. The increase in Caspase-3, Bax, and 8-OHdG positivity indicates that increased cell death and oxidative DNA damage contribute to this illness. Additionally, there is evidence of endoplasmic reticulum stress, as shown by the increase in CHOP and GPR78 positivity. Undoubtedly, the scientific literature recognizes the gut as a catalyst for the development of numerous organ dysfunctions [2]. The ischemic intestine generates free radicals and inflammatory mediators that damage remote organs, including the liver, kidney, lung, and heart, via blood circulation and lymphatic drainage [1]. The liver’s portal system, in particular, is the primary and most commonly damaged organ in this illness. Simultaneously, this condition impacts the liver, leading to the entry of these inflammatory mediators into the systemic circulation, thus affecting other organs. The findings of our study indicate that intestinal I/R not only induces liver injury characterized by the necrosis of hepatocytes, inflammation of liver lobules, congestion of blood vessels, and elevated levels of hepatic enzymes but also impairs renal function, as shown by the elevated levels of creatinine. This data additionally indicates that ERS plays a role in the development of liver damage caused by intestinal I/R.

Our biochemical examination revealed that the levels of creatine, AST, ALT, and TBARS increased in the I/R group. However, these increases were reduced in the TMZ and DEX groups. Additionally, the levels of TT increased in these groups. However, only the TMZ group presented a notable reduction in creatine levels, indicating that the renoprotective effect of TMZ may be more prominent. These findings indicate that the metabolic impacts of TMZ may be more noticeable, particularly on kidney function. This finding aligns with multiple studies documented in the literature [5,41,42]. The impact of DEX on creatine levels was insignificant, indicating that the renoprotective effect of DEX is not as substantial as that of TMZ. The histopathological and IHC results of our investigation revealed that both the TMZ and DEX treatments effectively decreased I/R damage. However, there are notable distinctions between the two therapies. The administration of TMZ was found to decrease intralobular and perilobular necrosis and vascular congestion. However, this decrease was less significant than that resulting from the administration of DEX. In contrast, DEX therapy has shown a superior ability to protect liver tissue by lowering intralobular and perilobular necrosis, vascular congestion, and inflammatory cell infiltration. This data indicates that the anti-inflammatory and antioxidant properties of DEX may be more potent than those of TMZ. Moreover, our biochemical and histological findings indicate a stronger protective effect of TMZ on the kidney and DEX on the liver. These findings suggest that these drugs may have distinct effects on different organs. Nevertheless, these disparities can also be explained by the impact of DEX on the central nervous system through alpha-2 adrenergic receptors. The calming and analgesic properties of DEX enhance its efficacy in diminishing inflammation and oxidative stress. Furthermore, this research strongly supports the efficacy of DEX in lowering oxidative stress and inflammation, as has been evidenced [10,13,43]. Despite being older and less common, studies have examined the hepatoprotective impact of TMZ in hepatic I/R [44,45].

During the initiation of apoptosis, the proapoptotic protein Bax is upregulated, whereas the antiapoptotic protein Bcl-2 is downregulated [46]. The expression levels of caspase-3, Bax, and Bcl-2 in rat liver tissues were assessed via immunohistochemical labeling. Apoptosis seems to occur in the I/R-induced rat liver injury model, with the caspase-3/Bax/Bcl-2 pathway contributing to the apoptotic process. The IHC analyses indicated that apoptosis in I/R-induced rat liver injury was markedly reduced by I/R+TMZ and I/R+DEX pretreatment, with considerable suppression of Bax-2. The antiapoptotic effect of DEX appears to be more pronounced than that of TMZ. Research has also revealed the inhibitory effect of DEX on apoptosis [47]. Chen et al. demonstrated that DEX effectively decreased apoptosis in cases of cardiac ischemia–reperfusion injury [10]. Moreover, other investigations have documented the antiapoptotic properties of DEX in different organs [9,10,23,47,48,49,50,51,52,53]. Dexmedetomidine, an alpha-2 adrenergic receptor agonist, has antiapoptotic effects by inhibiting the mitochondrial apoptosis pathway [50]. This transpires through the modulation of Bcl-2 family proteins (notably elevating Bcl-2 and diminishing Bax), hence reducing mitochondrial membrane permeability and obstructing cytochrome c release [50]. Moreover, its antioxidant effects mitigate oxidative stress, hence inhibiting apoptotic signals. Trimetazidine, however, inhibits apoptosis by maintaining cellular energy and mitigating oxidative damage [54]. Nonetheless, it may not be as efficacious as dexmedetomidine in directly influencing mitochondrial apoptotic processes. Trimetazidine stabilizes cellular energy metabolism, whereas dexmedetomidine specifically targets antiapoptotic pathways.

In our study, the assessment of necrosis via histopathological analysis and apoptosis via immunohistochemical analysis, along with the observed increase in both instances following damage, may have been contradictory; however, this is not the case. Instead, it reflects the evaluation of complementary biological processes via distinct techniques. Necrosis is a form of passive, uncontrolled cellular death that typically arises from acute injury, ischemia, or toxic influences. It is characterized by cellular edema, membrane lysis, and inflammation. This condition can be readily identified through morphological alterations, including tissue architectural degradation and coagulative necrosis, in histological evaluations such as H&E staining. Apoptosis is defined by biochemical and morphological characteristics, including cell shrinkage, chromatin condensation, and DNA fragmentation, and signifies a systematic process of cellular death [55]. Apoptosis is identified via cleaved caspase-3 or TUNEL staining, which indicates the presence of apoptotic signals prior to cellular death. Neurosis and apoptosis are invariably not distinct processes. In ischemia–reperfusion injury, cells may initially undergo apoptosis, and if this process remains unresolved, subsequent necrosis may ensue. Consequently, histological studies can identify necrotic cells in the terminal phase, whereas immunohistochemistry analysis can reveal apoptotic processes prior to their progression to necrosis [56]. Another crucial element is the timing of tissue analysis. Apoptosis may transpire early in the injury process, whereas necrosis may manifest later when cells have endured permanent damage. Consequently, apoptotic markers may be identified immunohistochemically despite the presence of necrotic characteristics observed via histological examination. Immunohistochemistry may exhibit greater sensitivity and specificity in identifying early apoptotic processes, whereas histopathology assesses a broader spectrum of tissue damage, including necrosis. The literature indicates that both apoptosis and necrosis occur in ischemia injury models, contingent upon the extent of cellular damage and the capacity of the tissue to recover. Research indicates that apoptotic and necrotic pathways can coexist, with apoptosis serving as the initial response and necrosis becoming more evident when cellular damage reaches an irreversible stage [56]. In conclusion, the apparent discrepancy between the two tests is not a true contradiction but instead illustrates the intricate and evolving nature of cell death. The simultaneous application of both techniques yields a more thorough evaluation of the cellular response by documenting early apoptotic alterations (immunohistochemical) and subsequent necrotic consequences (histopathological).

Our study assessed 8-OHdG as a marker for oxidative DNA damage. The I/R group presented a significant increase in the number of 8-OHdG-positive cells, which was dramatically reduced with TMZ and DEX treatments. Specifically, DEX has been found to be more efficient at decreasing indicators of oxidative stress. Multiple studies in the scientific literature have demonstrated that DEX mitigates oxidative stress by enhancing the body’s antioxidant defense mechanisms [57,58,59].

The primary focus of our work was on the expression of CHOP and GRP78, which are essential indicators of ERS. The results of our study indicate that both drugs effectively decrease the number of hepatocytes positive for CHOP and GRP78, which are known to increase due to I/R injury. Nevertheless, the impact of DEX on these indicators is more potent than that of TMZ.

CHOP is recognized as a proapoptotic factor that is triggered in response to ERS [22]. Our investigation revealed that the control group had no hepatocytes positive for CHOP, whereas the I/R group presented a notable increase in CHOP-positive hepatocytes. This discovery provides evidence that ERS plays a crucial role in I/R injury: the administration of TMZ and DEX therapies led to notable decreases in hepatocytes that were positive for CHOP. Specifically, the decrease in CHOP-positive cells resulting from DEX compared with TMZ indicates that DEX diminishes ERS-triggered apoptosis. The literature extensively explores the involvement of ERS and CHOP in I/R injury [23,25,60]. Zhang et al. reported that DEX inhibits CHOP production by decreasing ER stress, resulting in protective effects on the liver [23]. While the impact of TMZ on ERS and CHOP has received limited research attention, the metabolic effects of TMZ and its ability to regulate oxidative stress may account for the reported decrease in CHOP in this study [61]. 

GRP78, a chaperone protein, facilitates protein folding in the ER and is utilized as an indicator of ER stress. GRB78-positive hepatocytes were not present in the control group but were considerably increased in the I/R group. The administration of TMZ and DEX led to substantial decreases in the number of hepatocytes positive for GRP78. Specifically, the number of hepatocytes positive for GRP78 was lower in the I/R+DEX group than in the I/R group. The role of GRP78 in ERS is widely supported in the literature [18,21,22,60]. For example, Zhang and colleagues provided evidence that DEX mitigates ERS by decreasing the expression of GRP78 [23]. Moreover, the ability of DEX to reduce oxidative stress and inflammation may contribute to the reduction in GRP78-positive cells. While the impact of TMZ on GRP78 remains uncertain, this compound may also contribute to proteostasis by alleviating ERS [61].

The biological mechanisms responsible for the reduction in oxidative stress and ERS caused by DEX are linked to the activation of alpha-2 adrenergic receptors [23,62]. DEX is renowned for its anti-inflammatory and antioxidant characteristics, which are likely crucial in diminishing ERS. Specifically, the calming and pain-relieving properties of DEX can help maintain a stable internal environment in the ER by regulating how cells respond to stress. Moreover, previous research has indicated that DEX controls the body’s response to oxidative stress by activating Nrf2 pathways, reducing ERS [51,63]. In contrast, TMZ mitigates cellular stress by controlling energy metabolism and mitochondrial activities [7,64,65,66]. The antioxidative characteristics of TMZ may contribute to the reduction in oxidative stress and thereby alleviate ERS. Nevertheless, the impact of TMZ on ERS remains ambiguous, necessitating further investigation in this area.

The beneficial effects of TMZ and DEX in protecting against I/R injury provide strong support for the therapeutic application of these drugs. The heightened potency of DEX may explain its preference in critical care and surgical procedures. Nevertheless, it is advisable to remember some limitations when interpreting this study. Initially, this study was carried out on a controlled model, and additional investigations are needed to extrapolate the results to real-world medical environments. In our work, TMZ was administered seven days before the induction of the I/R model. Nevertheless, ischemia situations frequently lack clinical predictability. From this standpoint, more research is necessary to examine the impact of the timing of TMZ use in early interventions. In our work, we exclusively utilized male rats. However, further investigations are needed to assess the impact of sex variations on the observed effects. The limited sample size may have hindered the detection of significant differences through biochemical variations. The optimal pharmacological dose and duration of treatment have not been determined. Consequently, studies examining the effects of varying doses and durations may yield divergent outcomes. Furthermore, this study did not determine whether combining both drugs resulted in a synergistic effect. Under such circumstances, concurrently administering both medications may yield greater efficacy in mitigating liver and kidney damage. The histological study did not include renal tissue, which restricted our ability to assess renal damage further. Insufficient mechanistic investigations have impeded a comprehensive elucidation of the molecular-level mechanisms of action. It is imperative to design more extensive studies that account for all these limitations in future studies.

## 5. Conclusions

To summarize, this study verified that intestinal I/R leads to liver injury, whereas the drugs TMZ and DEX mitigate the damage caused by hepatic I/R. Furthermore, this study demonstrated that DEX has more potent protective effects than TMZ does. Specifically, DEX exhibited a more significant enhancement than TMZ did in terms of histological observations. These findings support the utilization of these substances in therapeutic settings and provide insight for future investigations. Nevertheless, considering the limitations of this study, further extensive and meticulous investigations are necessary.

## Figures and Tables

**Table 1:** Results of the biochemical analysis (median, 25–75% interquartile range).

	Creatinine (mg/dL)	AST (U/L)	ALT (U/L)	TBARS (mmol/L)	TT (mmol/L)
Control	0.28 (0.26–0.30)	126 (119–145)	67.5 (61–78)	1.00 (0.52–1.94)	0.45 (0.28–0.70)
I/R	0.59 (0.54–0.68) ^a^	368 (205–547) ^a^	158 (92–198) ^a^	2.32 (1.69–2.69) ^g^	0.26 (0.23–0.30) ^i^
TMZ	0.29 (0.26–0.32) ^b^	263 (228–269) ^d^	119 (72–134)	2.15 (1.85–2.46)	0.25 (0.22–0.28) ^j^
DEX	0.44 (0.31–0.73) ^c^	255 (237–585) ^e^	102 (87–183) ^f^	2.35 (1.69–3.45) ^h^	0.26 (0.25–0.31)
*p* value (between groups)	<0.001 *	<0.001 *	<0.001 *	0.007 *	0.005 *

Intergroup comparisons were performed via the Kruskal–Wallis test followed by pairwise Mann–Whitney U test comparisons (Dunn–Bonferroni correction). AST: aspartate aminotransferase, ALT: alanine aminotransferase, TBARS: thiobarbituric acid reactive substances, TT: total thiol, I/R: ischemia–reperfusion, TMZ: trimethylazidine, DEX: dexmedetomidine; ^a^ *p* < 0.001, control-I/R; ^b^ *p* = 0.006, TMZ-I/R; ^c^ *p* = 0.027, control-DEX; ^d^ *p* = 0.033, control-TMZ; ^e^ *p* = 0.014, control-DEX; ^f^ *p* = 0.039, control-DEX; ^g^ *p* = 0.018, control-I/R; ^h^ *p* = 0.034, control-DEX; ^i^ *p* = 0.038, control-/I/R; ^j^ *p* = 0.008, control-TMZ. * *p* ≤ 0.05 is significant.

**Table 2:** Liver Histopathological Damage Score (LHDS) values (median, 25–75% interquartile range).

Group	Hydropic Degeneration of Hepatocytes	Intralobular Necrosis	Interlobular Necrosis	Perilobular Inflammation	Vascular Congestion	LHDS
Control	0(0-0)	0(0-0)	0(0-0)	0(0-0)	0(0-0)	0(0-1)
I/R	2(2-2) ^a^	2(2-2) ^a^	2(2-2) ^a^	1(1-1) ^a^	2(1-2) ^a^	9(8-10) ^a^
I/R+TMZ	1(0-1) ^a,b^	1(1-1) ^a,b^	1(0-1) ^a,b^	1(0-1) ^a^	0(0-1) ^a^	4(3-5) ^a,b^
I/R+DEX	0(0-1) ^b^	0(0-1) ^b,c^	0(0-1) ^b,c^	0(0-1) ^d^	0(0-1) ^d^	2(1-3) ^e^

^a^ *p* = 0.001 compared with the control group, ^b^ *p* = 0.001 compared with I/R group, ^c^ *p* = 0.002 compared with I/R+TMZ group, ^d^ *p* = 0.015 compared with I/R+TMZ group, ^e^ *p* = 0.005 compared with control group, Kruskal–Wallis/Tamhane T2 Test.

**Table 3:** Semiquantitative analysis (median, 25–75% interquartile range).

Group	Caspase-3 Positivity Score	Bax Positivity Score	Bcl-2 Positivity Score	8-OHdG Positivity Score	CHOP Positivity Score	GRP 78 Positivity Score
Control	0(0-0)	0(0-0)	0(0-0)	0(0-0)	0(0-0)	0(0-0)
I/R	1(1-2) ^a^	2(2-3) ^a^	0(0-1)	2(2-3) ^a^	2(1-2) ^a^	2(2-2) ^a^
I/R+TMZ	0.5(0-1) ^b^	1(1-1.5) ^a,c^	1(1-2) ^a,c^	1(1-2) ^b^	1(0-1) ^b,e^	1.5(1-2) ^a.g^
I/R+DEX	0(0-0) ^b^	0(0-1) ^c,h^	2(2-3) ^a,c,i^	1(0-1) ^b,c,d^	0.5(0-1) ^b,f^	1(0-1) ^b^

^a^ *p* = 0.001 compared with control group, ^b^ *p* = 0.002 compared with control group, ^c^ *p* = 0.001 compared with I/R group, ^d^ *p* = 0.001 compared with I/R+TMZ group, ^e^ *p* = 0.023 compared with control group, ^f^ *p* = 0.013 compared with control group, ^g^ *p* = 0.007 compared with I/R group, ^h^ *p* = 0.01 compared with TMZ group, ^i^ *p* = 0.001 compared with TMZ group, Kruskal–Wallis/Tamhane T2 Test.

## Data Availability

All the data generated or analyzed during this study are included in this article. The datasets in the current study are available from the corresponding author upon reasonable request.

## Supplementary Materials

The following supporting information can be downloaded at https://www.mdpi.com/article/10.3390/biomedicines12102299/s1: Table S1: Liver Histopathological Damage Score (LHDS); Table S2: Semi-quantitative analysis (immunopositivity score).
