# Supplementary material for: Protective Effects of Trimetazidine and Dexmedetomidine on Liver Injury in a Mesenteric Artery Ischemia–Reperfusion Rat Model via Endoplasmic Reticulum Stress
Source: Biomedicines. 2024 Oct 10;12(10):2299. doi: 10.3390/biomedicines12102299 (PMC11504293; doi:10.3390/biomedicines12102299)
Supplement: Supplementary file 1 [file biomedicines-12-02299-s001.zip › biomedicines-3192624-supplementary.pdf]

Table S1. Liver Histopathological Damage Score (LHDS).

Severity	Description	Score
Hydropic Degeneration of Hepatocytes		
**Absent**	≤5%	0
**Mild**	≤25%	1
**Markedly**	≤50%	2
**Severe**	≤75%	3
Intralobular Necrosis		
**Absent**	≤5%	0
**Mild**	≤25%	1
**Markedly**	≤50%	2
**Severe**	≤75%	3
Interlobular Necrosis		
**Absent**	≤5%	0
**Mild**	≤25%	1
**Markedly**	≤50%	2
**Severe**	≤75%	3
Perilobular Inflammation		
**Absent**	≤5%	0
**Mild**	≤25%	1
**Markedly**	≤50%	2
**Severe**	≤75%	3
Vascular Congestion		
**Absent**	≤5%	0
**Mild**	≤25%	1
**Markedly**	≤50%	2
**Severe**	≤75%	3

Table S2. Semi-quantative Analysis (Immune-Positivity Score).

Severity	**Description**	**Score**
	Immun-Positivity Hepatocytes	
Absent	≤5%	0
Mild	≤25%	1
Markedly	≤50%	2
Severe	≤75%	3
